# Consequences of rewetting and ditch cleaning on hydrology, water quality and greenhouse gas balance in a drained northern landscape

**DOI:** 10.1038/s41598-023-47528-4

**Published:** 2023-11-18

**Authors:** Hjalmar Laudon, Virginia Mosquera, Karin Eklöf, Järvi Järveoja, Shirin Karimi, Alisa Krasnova, Matthias Peichl, Alexander Pinkwart, Cheuk Hei Marcus Tong, Marcus B Wallin, Alberto Zannella, Eliza Maher Hasselquist

**Affiliations:** 1 Department of Forest Ecology and Management, Swedish University of Agricultural Sciences, Umeå, Sweden; 2 Department of Aquatic Sciences and Assessment, Swedish University of Agriculture Sciences, Uppsala, Sweden

**Keywords:** Environmental sciences, Hydrology, Biogeochemistry

## Abstract

Drainage for forestry has created ~ 1 million km of artificial waterways in Sweden, making it one of the largest human-induced environmental disturbances in the country. These extensive modifications of both peatland and mineral soil dominated landscapes still carry largely unknown, but potentially enormous environmental legacy effects. However, the consequences of contemporary ditch management strategies, such as hydrological restoration via ditch blocking or enhancing forest drainage to promote biomass production via ditch cleaning, on water resources and greenhouse gas (GHG) fluxes are unclear. To close the gap between science and management, we have developed a unique field research platform to experimentally evaluate key environmental strategies for drained northern landscapes with the aim to avoid further environmental degeneration. The Trollberget Experimental Area (TEA) includes replicated and controlled treatments applied at the catchment scale based on a BACI approach (before-after and control-impact). The treatments represent the dominant ecosystem types impacted by ditching in Sweden and the boreal zone: (1) rewetting of a drained peatland, (2) ditch cleaning in productive upland forests and (3) leaving these ditches unmanaged. Here we describe the TEA platform, report initial results, suggest ways forward for how to best manage this historical large-scale alteration of the boreal landscape, as well as warn against applying these treatments broadly before more long-term results are reported.

## Introduction

Wetlands are among the most degraded ecosystems globally, and are in many places in urgent need of restoration in order to regain their natural characteristics and re-establish their physical, chemical, and biological functions^1^. At northern latitudes, most wetlands are peatlands with seasonal water logged conditions, long-term carbon sequestration functions, and unique biodiversity. Historically, extensive peatland areas have been drained for agricultural or forestry purposes, resulting in many peatlands ceasing to be wetlands as the ditch-networks effectively removed the water logged conditions^2^. Moreover, many wet upland forests on mineral soil have been ditched in the boreal region to increase tree growth^3^. In Sweden, the majority of ditches in the forest landscape were dug during the first half of the 1900s, resulting in a total of approximately 1 million km of a century old artificial drainage channels today^4^. Currently, this network of ditches is one of the most widespread human-induced environmental disturbances with largely unknown, but potentially enormous, legacy effects on soils, waters, and biodiversity. After Finland and Russia, Sweden has the most drained forested land in the world^5^, covering the entire country, except for the high mountain areas bordering Norway. Currently, drained peatlands make up over 14% of the productive forest in Sweden^6^, but large drained areas have also remained unproductive, primarily because of nutrient limitations.

In Sweden, the decision on what to do with the large number of historical drainage ditches remains an open question: hydrological restoration by ditch blocking to bring back more pristine environmental conditions, increase ditch cleaning activities to maintain the potential for forest biomass production, or leave them unmanaged? Before any informed management decision can be made, improved understanding about what the specific implications of the different options entails addressing several critical scientific challenges. Hydrological restoration, i.e. raising the groundwater levels and, thus, rewetting drained organic soils to conditions considered more natural has become a highly politicized issue with large amounts of governmental subsidized funds available for rewetting in many countries—primarily with the goal of mitigating climate change. Yet, the science underpinning the desired outcomes of peatland rewetting is largely lacking, at least for the time frames relevant for alleviating the climate crisis. In fact, there is limited empirical evidence for the climate mitigation potential of peatland restoration is as it can have negative effects on the greenhouse gas (GHG) balance due to the increased emissions of methane (CH_4_), which is a GHG with > 80 times larger global warming potential than carbon dioxide (CO_2_) over a 20-years period^7^. In Europe, only few published short-term studies exist from restored peatland forests in Finland, which observed reduced CO_2_ emissions^8^, but up to 20 times increased CH_4_ emissions^9^. Meanwhile, such empirical data are currently entirely missing from Sweden, particularly in the boreal region where drained nutrient-poor peatland forests dominate the landscape. Instead, our understanding of GHG emissions from hydrologically restored peat soils primarily relies on studies from rewetted peat extraction areas^10,11^, undrained pristine peatlands or models^11^. Thus, the actual GHG mitigation potential of restoration measures is at present highly uncertain. Of course, there are other reasons to rewet peatlands, but evidence does not always support that rewetting is best for alleviating impacts on hydrology^12^, nor that it is beneficial for water quality^13,14^ or even biodiversity^15,16^.

When ditches degrade because of sediment infilling and vegetation establishment, ditch cleaning (DC) can improve the survival of new tree seedlings and forest productivity by lowering groundwater levels^17^. In fact, some wet upland forest areas on mineral soil require ditch cleaning after clear-cutting to allow reestablishment of new seedlings. The water table typically rise after forest harvest due to the loss of evapotranspiration that maintains low groundwater levels. Similar to peatland restoration, there is limited empirical evidence on the environmental consequences of ditch cleaning that can be used to evaluate the effects on both water resources and the carbon balance, at least in the Swedish context^18^. However, studies in Finland have demonstrated that ditch cleaning can be a major source of sediments and nutrients downstream^19,20^, and in Estonia it has been shown to decrease habitat availability, abundance and diversity of aquatic invertebrates^21^. Furthermore, ditch cleaning also results in increased mineralization of soil organic carbon, which has negative consequences for GHG emissions^22,23^. However, the potential for enhanced tree growth following ditch cleaning increases the rate of CO_2_ uptake, which is neglected in most evaluations. In Sweden, empirical data for ditch cleaning effects on GHG fluxes are currently limited to the short-term responses during the initial post-treatment years in forest clear-cuts on drained upland mineral soils^24^ and peatlands^25^, suggesting highly site-specific responses. Despite uncertainties surrounding the consequences of ditch cleaning, it has been suggested that Sweden should clean ditches on approximately 400,000 ha of drained land to maintain current forest production levels^6^.

Given the limited scientific understanding on both restoration and ditch cleaning, the risk with applying these management actions broadly without proper evaluation is that they might enhance the negative consequences on both GHG emissions and water resources. Therefore, to overcome the limited knowledge about different management strategies of drained areas, our goal was to evaluate how different management actions within historically drained boreal landscapes influence water quantity and quality, as well as the carbon balance. Here, we report from the 4 years of our study at the Trollberget Experimental Area (TEA) in northern Sweden. This unique research infrastructure provides the first fully replicated ditch management experiment applied at the catchment scale in Sweden allowing the evaluation of water quantity, quality and carbon balance consequences of drained forests that have been (1) restored, (2) ditch cleaned, and (3) left unmanaged.

## Material and methods

### The TEA experimental infrastructure

**Figure 1:**
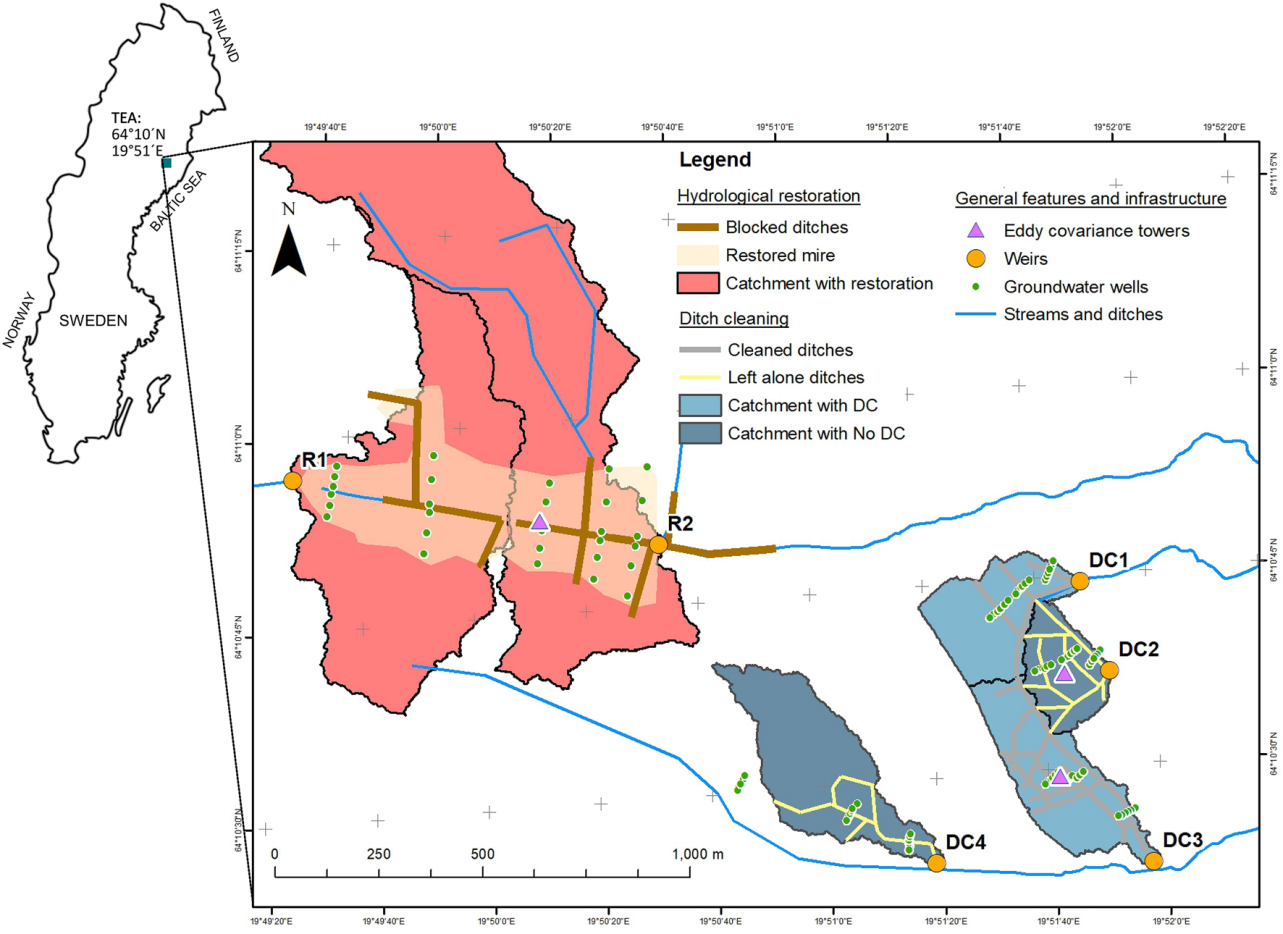
Trollberget Experimental Area (TEA) catchments with an insert of Sweden (left) with monitoring locations. The bold grey, yellow and brown lines denote the cleaned, left alone and filled ditches, respectively; delineated blue and red shaded areas are the different treatment catchments; green circles are the locations of water quality monitoring sites and purple triangles are the locations of Eddy covariance towers. DC1-DC4 (in blue) are the ditch cleaned experimental sites and R1 and R2 (in red) are the hydrological restoration experimental sites. Map was generated using ArcMap 10.6^32^.

The Trollberget Experimental Area (TEA; 64° 10′ N, 19° 51′ E), is located approximately 50 km northwest of the city of Umeå in northern Sweden (Fig. 1). The research infrastructure is designed to test the effects of rewetting through ditch blocking and draining through ditch cleaning in historically drained boreal catchments in comparison to leaving the degraded ditch networks unmanaged. The TEA is built around replicated and controlled experimental catchments based on a BACI (before-after control-impact) approach. The TEA infrastructure is part of the Svartberget research station that includes the long-term research facilities of the Krycklan Catchment Study (KCS, 64° 25′ N, 19° 46′ E)^26^ and the Kulbäcksliden Research Infrastructure (KRI, 64° 18 N, 19° 56′ E)^27^, which altogether provide long-term study sites in upland forests, drained peatland forests and natural mires, which serves as controls. The open peatlands in the area are oligotrophic minerogenic mires, dominated by *Sphagnum* spp. together with sparse coverage of sedges and dwarf shrubs with some slow growing pine trees. The forested catchments are covered by Scots pine (*Pinus sylvestris* L.) trees and Norway spruce (*Picea abies* L*.*) primarily with an understory of ericaceous shrubs, mostly bilberry (*Vaccinium myrtillus*) and lingonberry (*Vaccinium vitis-idaea*) on moss-mats of *Hylocomium splendens* and *Pleurozium schreberi*. Here, the underlying soils are dominated by Humic Podzol, with some Humo-Ferric Podzol in drier areas and Histosols in wet. The climate is typical of the boreal zone, characterized as a cold temperate humid type with relatively short and cool summers followed by long dark winters. On average the catchments are snow covered during 167 days typically from late October to early May^28^. Mean annual air temperature is 2.1 °C (30 years mean, 1986–2015) with the highest mean monthly temperature in July and the lowest in January (+ 14.6 and − 8.6 °C, respectively^29^). Total annual precipitation averages 614 mm yr^−1^ of which approximately 35–50% falls as snow and 311 mm becomes runoff^30^.

The TEA infrastructure (Fig. 1, Table S1) consists of six treated experimental catchments with drainage areas ranging from 4 to 60 ha. Areas of study catchments were modelled using the Deterministic 8 (D8) algorithm^31^ based on a 2 × 2 m DEM in which we first burned the ditches into the DEM to the depth of 0.5 m (Whitebaox GAT 3.3). Two catchments (R1 and R2) were used for peatland hydrological restoration, where peat depth averaged 241 cm (range from 22 to 599 cm; n = 190) with an average top soil C/N of 42.7. Alongside, four catchments (DC1–DC4) with forest ditch cleaning management (DC 1 and 3) and without (DC2 and 4) on podzols, with pockets of histosols with an average organic soil depth of 140 mm (range from 80 to 260 mm). The rewetting or hydrological restoration (hereafter called “restoration”) was conducted in November of 2020 through conventional authority defined methods, using 20-ton crawling excavators taking on-site peat and trees to fill in the man-made ditches. The sparse tree canopy (basal area = 2.6 m^2^ ha^−1^) previously existing on the restored peatland site was cut and removed (unless used as filling material), leaving only small amounts of slash behind. In the ditch cleaned (DC) areas (Fig. 1), all trees in DC1-4 were clear-cut harvested (between August 2020 and February 2021). All logs were removed from the site, leaving slash behind to avoid driving damage to the soil. Harvesting was followed by ditch cleaning at two of the four catchments in September 2021 where a 20-ton crawling excavator were used to clean the ditches of DC1 and DC3, while, as references, in DC2 and DC4 the ditches were left to develop freely^26^. Typical ditch dimensions before cleaning at TEA was similar across the ditch cleaning catchments, on average 500 mm (± 150) deep and 1470 mm (± 360) wide at the top. This is comparable to ditches on the natural forest reference sites, that are on average 500 mm (± 210) deep and 860 mm (± 530) wide. For the two restoration area outlets (R1 and R2), the nearby, long-term natural mire research catchment C4 in KCS^26^ and C18 at the KRI Degerö Stormyr^27^ were used as controls to evaluate the effect of restoration on achieving natural conditions for hydrology and water quality analysis. For GHG flux measurements, the Hälsingfors drained peatland forest at KRI was used as reference^25^ to evaluate the effect of rewetting a drained peatland.

### Land-use history

Prior to the early 1900’s, peatlands constituted an important resource for farmers in Sweden. In northern Sweden, including the region of TEA, this meant that most mires were used for harvesting hay^2^. In the early to mid-1800’s, the forest logging frontier arrived to the region, which resulted in large areas being harvested and the quest for new forest management practices begun^33^. As a consequence, most low productive peatlands and areas with wet mineral soils were drained primarily by hand to enhance forest production^2^. In TEA, this was initially done around 1905, with a subsequent second wave of drainage activity in the 1930’s. In a recent analysis across 11 forest landscapes across Sweden (including the KCS, adjacent to TEA), Paul et al.^34^ showed that of the present channel network only 13% are natural streams, while the rest consist of ditches related to forest areas (56%), agricultural fields (6%) and roads (25%).

### Field measurements and analytical methods

Starting November 2018, pre-treatment hydrology [soil water table depth (WTD)], and stream water quality [including dissolved organic carbon (DOC), total dissolved nitrogen (TDN), total phosphors (TP), total mercury (THg) and total suspended solids (TSS)] were measured at the outlets of the six treated catchments. The same protocols and sampling intensities were followed in the six experimental catchments as within the KCS and KRI references. Terrestrial GHG flux measurements (incl. CO_2_ and CH_4_ exchanges) on the restored area started with the manual chamber technique in June 2018 and Eddy Covariance (EC) technique in January 2021. The pre-restoration EC measurement period, started in November 2019 and continued until the end of October 2021 and for the post-restoration period; data was analyzed data until November 2022. For the ditch cleaning area, the pre-ditch cleaning period ranged from late August 2020, after clear-cut activities were finished, until mid-September 2021, when ditch-cleaning activities started. Further, post-ditch cleaning period begun in late September of 2021 and lasted until the end of November 2022. The same procedures and protocols were used during the pre-treatment and post-treatment periods for both the terrestrial and aquatic programs.

#### Hydrology

At the peatland sites, WTD measurements were based on 30 dipwells (fully perforated and screened, extending into the underlying mineral soil up to 6 m below the ground surface). While half of the wells were continuously monitored for WTD using data loggers (Solinst Levelogger 5), the remaining were measured manually approximately every 2 weeks during the snow-free season. Similarly, in the ditch cleaning catchments, WTD were measured using 72 deep wells, with one-third continuously monitored (Solinst Levelogger 5), while the remaining were measured manually approximately every other week during the snow-free season. For the pre- and post-restoration period, the daily continuous data were analyzed, with 173 and 344 observations per sampling period, respectively. Further, for the pre and post-ditch cleaning period, daily continuous data were analyzed, with 237 and 204 observations per sampling period, respectively. At all sites, WTD was measured relative to the ground surface.

#### Water quality

The sampling program was flow adjusted, following the KCS and KRI laboratory and sampling frequency protocols^26,27^. During high flow-spring flood periods, samples were collected twice per week, during intermediate flow conditions associated with the growing season samples were collected every 2 weeks, and during winter base-flow once per month. For the pre- and post-ditch cleaning period, samples were collected from 16 to 35 and 11 to 24 observations per sampling period, respectively. For the pre- and post-restoration period, we sampled 26–37 and 22–44 observations per sampling period, respectively. Variability in the sampling occasions was due to that the streams dried out during the summer period.

The stream water samples were collected in acid-washed high-density polyethylene bottles and kept dark and cool during transport and storage. Samples were filtered (0.45 µm mixed cellulose ester (MCE) syringe filters, Millipore®) within 24 h and kept refrigerated at 4 °C until analysis (< 7 d after filtering). DOC analysis consisted of acidification of the sample for removing inorganic carbon, followed by combustion using a Shimadzu TOC-VCPH^35^. Filtered and unfiltered subsamples were frozen (− 20 °C) immediately after subsampling and stored for later analysis of TDN and TP, respectively at the Swedish University of Agricultural Sciences Biogeochemical Analysis Laboratory. The analytical methods for TDN has been described in detail by Blackburn et al.^36^. TP was analyzed at ALS Scandinavia AB (Aurorum 10, SE97775 Luleå, Sweden), on a "ThermoScientific ELEMENT 2" using method ISO 17294-2:2016. Total suspended solid samples were collected at the same time as nutrients, in 500 mL bottles. These samples were stored frozen until they were thawed then filtered using pre-weighed glass fiber filters (Whatman grade) with filtration apparatus under suction, as in the 2540 Solids protocol^37^. After filtering, filters plus collected sample were dried at 105 °C, thereafter weighed, and the total mass of suspended solids was calculated. Finally, THg were sampled and analyzed using and ultra-clean sampling protocol. Single-use plastic gloves were used and water were collected in acid-washed Teflon bottles. Blank samples containing ultrapure water (Milli-Q type 1) were collected regularly to ensure that the bottles and sampling routines did not cause contamination. Samples were kept dark and cold during transport. At the laboratory, samples for THg analyses were preserved by suprapure nitric acid (HNO_3_). Analyses of THg were performed at the Swedish Environmental Research Institute (IVL) using Cold Vapour Atomic Fluorescence Spectroscopy (CV-AFC) following the US EPA standard method 1631 version E^38^.

#### Terrestrial carbon fluxes

The EC technique was used to quantify the ecosystem-scale GHG exchanges at the TEA restored and at the forest clear-cut sites DC2 (as control) and DC3 (as ditch cleaning treatment). The measurement setup included a Campbell CPEC306 closed-path system that combines a CO_2_/H_2_O gas analyzer with a sonic anemometer to estimate the net ecosystem exchange (NEE) of CO_2_. At the TEA restoration site, a LI-COR LI7700 analyzer provided additional information on the ecosystem-scale exchange of CH_4_.

Plot-scale chamber measurements of CO_2_ and CH_4_ fluxes were conducted along replicated transects in bi-weekly intervals during the growing season at each EC flux station. The measurement setup included transparent and opaque chambers connected to a portable GHG analyzer. The measurements were carried out at different distances relative to the drainage ditch and across natural as well as experimental vegetation removal (+ trenching) plots to provide additional information on the spatial variation and component fluxes from soil and vegetation. Furthermore, each EC station included a suite of meteorological sensors (air temperature, radiation balance, relative humidity, wind speed and direction), phenology cameras and replicated soil profiles for continuous measurements of soil temperature and moisture as well as WTD.

### Statistical analyses

For hydrology and water quality, at each sample occasion a difference in mean value of the two restored sites; R1 and R2, and the mean value of their reference sites; C4 and C18 were calculated. For GHG, the same calculation were conducted using the KRI Hälsingfors drained peatland forest as a reference. For hydrology and water quality in the DC sites, the same calculations were carried out for the two ditch cleaned sites; DC1 and DC3, and their references; DC2 and DC4. Finally, for the GHG, the difference was calculated between one ditch cleaned site DC3 and its reference, DC2. The effect of restoration and ditch cleaning was tested by comparing the relative difference in mean values from before to after, except for the results of GHG at the restoration site. In case of the latter, data from the KRI Hälsingfors drained peatland forest were used to represent the before conditions since EC flux data were not available from the rewetting site prior to rewetting. Whether the change from before to after the treatments were statistical significant or not was tested in a generalized linear mixed model (GLMM) with treatment (i.e. restoration or ditch cleaning) as a fixed factor. A repeated structure was applied to account for the multiple sample occasions. The treatment effects were considered significant when *p *< 0.05.

## Results

### Hydrology

**Figure 2:**
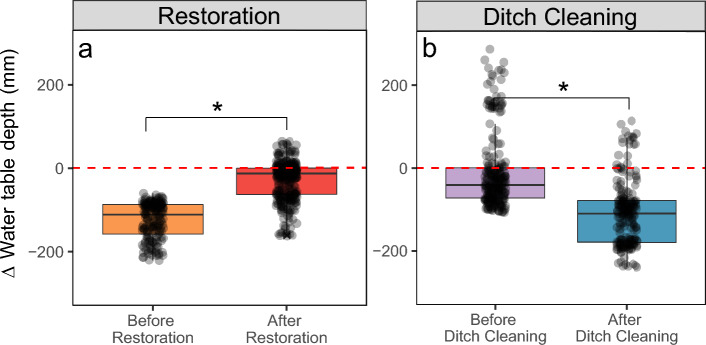
Differences in water table depth (WTD) between the treatment and control sites before and after interventions. The difference was computed as treatment minus control, thus positive values indicate that the WTD is greater at the treatment site than at the control site, while negative values indicate the opposite. Black circles represent individual data points. *denotes significant difference (*p* < 0.05) between treatments. Red dotted line denotes the control zero reference. Solid line in box plots is the median value, box extents are the interquartile range (IQR) and whiskers show the 1.5IQR value.

Restoration resulted in a statistically significant increase in the median WTD compared to the pre-restoration period (Fig. 2a). The median WTD at the restored sites rose from − 186 to − 77 mm after restoration, while the median WTD at the control sites increased by only 6 mm during the same time-period (from − 80 to − 73 mm), indicating that the median WTD was roughly the same during the pre- and post-treatment periods. Following ditch cleaning, the median WTD decreased significantly in relation to the control sites (Fig. 2b) from − 325 to − 430 mm.

### Water quality

#### Dissolved organic carbon

**Figure 3:**
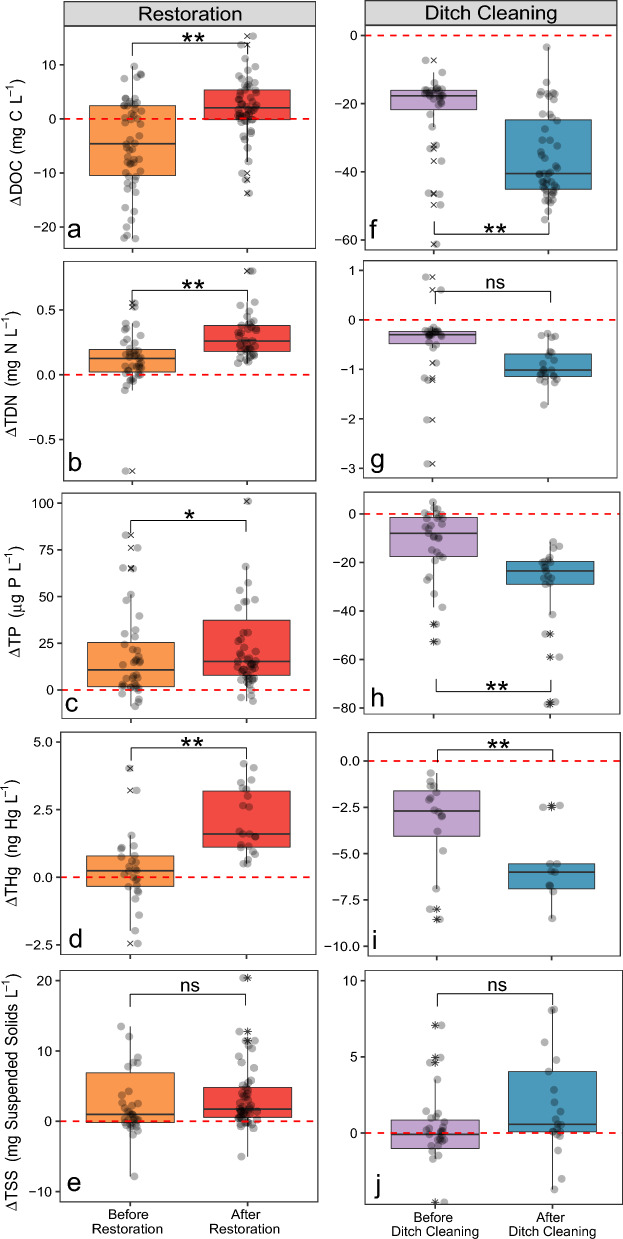
Differences between the treatment and control sites before and after interventions for DOC (**a** and **f**), TDN (**b** and **g)**, TP (**c** and **h**), THg (**d** and **i**) and TSS (**e** and **j**). The difference was computed as treatment minus control, thus positive values indicate that the solute is greater at the treatment site than at the control site, while negative values indicate the opposite. Black circles represent individual data points **denotes significant difference (*p* < 0.01) between treatments and *denotes significant difference (*p* < 0.05) between treatments, “n.s.” denotes no significant difference (*p* > 0.05) between treatments. Red dotted line denotes the control zero reference. Solid line in box plots is the median value, box extents are the interquartile range (IQR) and whiskers show the 1.5IQR value. Note that the scales for the y-axes show different magnitudes of concentrations.

The median DOC concentration in the restored sites (R1 and R2) increased significantly from 26.1 to 30.6 mg C L^−1^ after the hydrological restoration (*p* < 0.05), reaching above the average DOC concentration of the non-drained controls of 28.3 mg C L^−1^. In relation to the non-drained control sites, the DOC concentration of the restored sites increased by 6.7 mg C L^−1^ during the post-restoration period (Fig. 3a). For the dich-cleaning treatment at sites DC1 and DC3, the median DOC concentration increased by 4.6 mg C L^−1^, from 27.3 to 31.9 mg C L^−1^ after ditch cleaning (*p* < 0.05). However, in relation to non-treated controls (DC2 and DC4), the median DOC concentrations was lowered by over 22.8 mg C L^−1^ during the post-treatment period (Fig. 3f), as the non-treated controls increased from a median concentration of 42.6–69.5 mg C L^−1^ during the post-restoration period.

#### Nitrogen and phosphorus

The median TDN concentration in R1 and R2 increased significantly from 0.51 to 0.62 mg N L^−1^ after restoration (*p* < 0.05). Similarly, in relation to the non-drained control sites the restored sites increased by 0.11 mg N L^−1^ (Fig. 3b), as the non-treated control catchments decreased from a median concentration of 0.46–0.38 mg N L^−1^ during the post-restoration period. Conversely, the median concentration of total phosphorus (TP) for the restored sites decreased significantly (*p* < 0.05) from 31.5 to 20.2 μg P L^−1^ after restoration. However, in relation to the non-drained control sites the restored sites increased by 4.5 μg P L^−1^ (Fig. 3c), as the non-treated control catchments decreased in median TP concentration from 21.4 to 10.5 μg P L^−1^. For the ditch cleaning sites, the median TDN concentration increased from 0.61 to 0.97 mg N L^−1^ after treatment. However, in relation to the non-treated controls (DC2 and DC4), median TDN concentration was lower by 0.7 mg N L^−1^ (*p* < 0.05) post treatment (Fig. 3g), as the non-treated controls had an increase from 0.9 to 2.1 mg N L^−1^. Conversely, TP median concentration decreased significantly from 34.8 to 24.0 μg P L^−1^ after DC. In relation to the non-treated sites the median concentration of TP decreased (*p* < 0.01) by 15 μg P L^−1^(Fig. 3h), as the control catchments slightly increased in concentration from 41.1 to 46.5 μg P L^−1^ in the post-treatment period.

#### Mercury

The median THg concentrations in R1 and R2 increased significantly after restoration (5.7 ng L^−1^) compared to pre-treatment (4.1 ng L^−1^) (Fig. 3d). In contrast, in relation to the non-treated controls (DC2 and DC4) the median THg concentrations from the ditch cleaned sites, DC1 and DC3 were lower after treatment (Fig. 3i). This occurred despite the median concentrations were higher post (10.3 ng L^−1^) compared to pre-treatment (9.8 ng L^−1^), but the controls increased even more (from 12.9 to 16.0 ng L^−1^), indicating that other factors than ditch cleaning caused the increasing concentrations of THg.

#### Total suspended solids

The median difference in TSS concentrations between restored and non-drained control sites did not change after restoration (*p* > 0.05) at any flow conditions (Fig. 3e). However, measurements were more variable pre-treatment, with the pre-restoration drained sites (R1 and R2) having a median of 2.1 (± 16.4) mg L^−1^ and post-restoration of 1.9 (± 4.5) mg L^−1^. For the dich-cleaning treatment at sites DC1 and DC3, the median TSS concentration changed from 1.2 to 1.16 mg L^−1^ after ditch cleaning. However, in relation to non-treated controls (DC2 and DC4), the median TSS concentrations increased by 0.86 mg L^−1^ during the post-treatment period, but this was not statistically significant (*p* > 0.05, Fig. 3j), because the non-treated controls decreased from 1.53 to 0.54 mg C L^−1^ during post-treatment period. Moreover, the median difference in TSS between the treated (DC1 and DC3) and control catchments (DC2 and DC4) increased by about 670%.

### Terrestrial carbon fluxes

**Figure 4:**
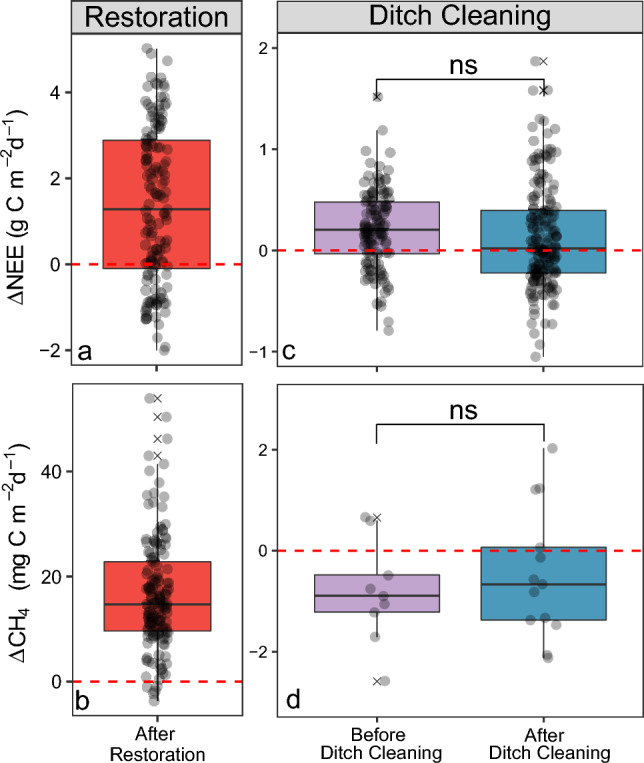
Difference in the daily sums of the ecosystem-scale exchange of CO_2_ (NEE, **a** and **c**) and methane (CH_4_; **b**) as well as plot-scale (CH_4_; Δ fluxes (**d**) over one growing season (mid-May to mid-October) following restoration (**a** and **b**) and before and after ditch cleaning (**c** and **d**). The difference is computed as treatment minus control. Black circles represent individual data points. “n.s.” denotes no significant (*p* > 0.05) difference between treatments. Red dotted line denotes the control zero reference. Solid line in box plots is the median value, box extents are the interquartile range (IQR) and whiskers show the 1.5IQR value. Note that the scales for the y-axes show different magnitudes of fluxes.

The peatland site acted as a net CO_2_ source in the first growing season following restoration while the nearby drained peatland forest control site acted as a net CO_2_ sink during the same period. The median of the daily NEE differences between both sites indicates that NEE at the restoration site was 2.0 g CO_2_–C m^−2^ d^−1^ higher than at the drained peatland forest (Fig. 4a). Similarly, the peatland restoration site acted as a source for CH_4_ while the drained peatland control site was a small sink for CH_4_. The median of the difference in daily CH_4_ fluxes between the two sites suggests that CH_4_ emissions increased by 15.0 mg CH_4_–C m^−2^ d^−1^ following restoration (Fig. 4b).

Both the ditch cleaned and control sites (DC3 and DC2, respectively) were sources of CO_2_ during the pre-treatment period, ranging (10th and 90th percentile) commonly from about 1.1 to 3.5 g CO_2_–C m^−2^ d^−1^ during mid-May to mid-October. The median of the daily NEE differences between both sites indicates that CO_2_ emissions at the treatment site (DC3) were about 0.20 g CO_2_–C m^−2^ d^−1^ higher than the control site (DC2) during the pre-treatment period (Fig. 4c). In the first growing season following ditch cleaning, the median of the daily NEE difference was 0.02 g CO_2_–C m^−2^ d^−1^. Given a median daily flux of 3.0 g CO_2_–C m^−2^ d^−1^ at DC3 in the pre-treatment growing season, this suggests a decrease of 6.3% due to ditch cleaning. Meanwhile, both experimental sites commonly acted as small sinks for CH_4_ with rates ranging from − 10 to − 4 mg CH_4_–C m^−2^ d^−1^. The median of the daily CH_4_ flux differences between treatment (DC3) and control (DC2) sites were − 0.39 mg CH_4_–C m^−2^ d^−1^ in the pre-treatment period, which increased by 2% to 0.58 mg CH_4_–C m^−2^ d^−1^ in the growing season following ditch cleaning (Fig. 4d).

## Discussion

### Hydrology

Analyzing the response of the WTD is an important aspect of understanding the effectiveness of restoration efforts. Our results showed that restoration significantly increased the median WTD within the first 2 years after rewetting compared to pre-restoration, while the control sites WTD median remained relatively stable across the entire experimental period. This indicates that restoration was successful in significantly raising the groundwater level back to pre-drained conditions. Our findings are aligned with similar case studies conducted in boreal ecosystems that focused on restoration impacts on water table^16,39–41^, as well as a with a large recent meta-analysis^12^.

Additionally, our results demonstrate that ditch cleaning was effective in lowering the WTD. Specifically, we observed a significant decrease of − 104 mm compared to the pre-treatment period. The results are in accordance with previous studies in boreal ecosystems^12,42,43^. For instance, Päivänen and Sarkkola^44^ examined how thinning and maintaining the ditch network affected the water table level in a Scots pine forest in southern Finland. Their findings indicated that thinning had a negligible impact on the water table, but ditch cleaning effectively counteracted any changes. It should be noted, however, that ditch cleaning does not necessarily always lead to a lower water table, but that the effectiveness may vary depending on the soil types^43^. Thus, conducting a comprehensive assessment is important to determine the necessity and effectiveness of ditch cleaning during forest management planning.

### Water quality

#### Dissolved organic carbon

The few existing previous studies regarding effects of wetland restoration on DOC in runoff for boreal conditions show somewhat divergent effects. The observed median increase in stream DOC concentration after restoration at TEA contradict observations from restored peatlands in Finland where peat pore water DOC generally decreased^14^. On the other hand, restoration of nutrient-rich fens in boreal Finland resulted in a five-fold increase in DOC concentration of runoff water during the first year but then leveled off during the following 5-year period^13^. Shallower groundwater levels after restoration enhances the connectivity to C-rich, and potentially more easily degradable terrestrial sources that historically became hydrologically disconnected by the drainage. The increased water volume after restoration could also lead to altered hydrological flow paths, which in turn affect the relative source contribution by different laterally and vertically distributed C pools within the peatland^45,46^. The elevated DOC concentrations found at TEA will have implications for downstream water quality as well as for C balance estimates of the restored wetland. Whether these effects will be long-lasting or level off over time will require continued monitoring and evaluation.

The ditch cleaning had a significant, but opposite effects compared to rewetting with decreasing DOC concentrations in relation to the left-alone ditches. Similarly, other ditch cleaning studies^47,48^ have also reported decreased DOC concentration with different extents depending on the characteristics of the surrounding catchment soils and the time scale analyzed^49,50^. The decreased DOC concentration in runoff following ditch cleaning at TEA are likely tightly linked to changes in WTD and dominating flow paths^51^. By lowering the hydrological flow paths, C-rich soil layers were avoided and hence resulted in lower concentrations of DOC. Increased export through mineral soil also affects the mobility of DOC as they act as sorption sites for negatively charged organic molecules^47^. The physical removal of vegetation and accumulated organic material in the ditch is likely another reason for the decreasing DOC levels following ditch cleaning. Nieminen et al.^52^ suggested that the ditch cleaning effect on runoff chemistry in general, and DOC specifically, could be linked to the degree ditch cleaning was made down to mineral soils.

#### Nitrogen and phosphorus

Forest streams have strong linkages to their terrestrial surroundings obtaining most of their N and P from adjacent landscapes^53^. Specifically, boreal forests in general tend to be N limited with limited losses to aquatic ecosystems^54^, while P exports seem to be more site specific. For both nutrients, natural long-term declining trends have been observed in boreal catchments^55^, however there is typically an increase in leaching after forest management^56^. Therefore, understanding the effect of ditch management on N and P exports is important in a managed boreal forest context. Here, we report an opposite response of nutrient concentrations draining restored and ditch cleaned catchments; TDN and TP concentrations in waterways increased after restoration and decreased after ditch cleaning.

Restoration significantly increased TDN in the first 2 years after treatment, corroborating observations from Finnish restored peatlands^57^. The increase in TDN, is likely related to the anoxic conditions caused by the increase in WTD and, thus, resulted in an increased mobilization and leaching of organic N^13^ or alternatively in an accumulation of mineralized ammonium (NH_4_) in soil water in the absence of nitrification^58^. Similarly, TP increased significantly after restoration and, although this is also in general agreement with the results of other re-wetting results^13^, the factors controlling the release of P in anoxic conditions is less clear^13^. Yet, the increase of both TDN and TP could have also been influenced by a reduced nutrient uptake by trees due to the harvest done before the ditch blocking^59^.

Similar to other studies^48,49,60^, the ditch cleaning did not affect TDN. However, these results could be an artifact of lumping the inorganic and organic forms of N into TDN^61^ as ditch cleaning could result in increasing levels of inorganic N^62^ and decreasing organic N^48^. Conversely, our results show that TP significantly decreased after ditch cleaning. Other studies demonstrate contradictory results for TP, as they have shown an increase in stream concentration^49^, slight decrease^63^ and no change^60^. These divergent results could be an effect of site specific conditions such as soluble P availability in the organic layer of the soils^64^ or the amount of adsorption capacity due to iron concentrations (Fe) or aluminum (Al) content of the peat^65^.

#### Mercury

Following the restoration, the change in THg largely followed that of DOC, likely as a consequence of flooding of peat soils that affected the hydrological connectivity between the surrounding landscape and stream. Most previous studies regarding peatland restoration effects on Hg have been focusing on the bioavailable form of Hg, methylmercury (MeHg). There is a risk of elevated MeHg formation by anaerobic microorganisms capable of Hg methylation when drained peat soils become transformed to aquatic habitats. Elevated MeHg concentrations in surface waters has been observed following large—scale experimental flooding^66^, building of beaver ponds^67,68^ and creation of artificial wetlands^69^. Although THg is less bioavailable, an increased mobilization could be a source of MeHg downstream and hence further enhance the already high Hg levels. The Hg concentrations in Sweden exceeds the EU Water Framework Directive environmental quality standard by up to 100 times across all water bodies in Sweden^70^, and are now at levels that the World Health Organization (WHO) deems potentially harmful for human consumption in around half of the Swedish inland waters^70,71^.

In contrast to the restoration experiment, ditch cleaning did not significantly increase the THg concentration in the drainage system. Instead, the concentrations of THg decreased in the post-treatment period in relation to the controls. The decrease in THg following the treatment can, similarly to DOC, be explained by changing flow-paths to deeper peat horizons with less easily mobilized Hg. Previously, only few existing studies have reported the effect of ditch cleaning on Hg in surface water. One such study conducted in southern Sweden^50^, found extremely high THg concentrations during the first couple of days after treatment, but after the initial sharp increase the concentration declined rapidly down to background levels. Other studies, focusing on MeHg, found no effect^72^. In the present study, we detect no significant increase in THg during the year following ditch cleaning, but also no initial increase, although the study included frequent sampling (up to twice a day) in the first weeks following ditch cleaning.

#### Total suspended solids

A recent review found that TSS is typically elevated the first 2 years after ditch cleaning compared to pre-treatment levels, and then stabilize over time^61^. Ditch cleaning results in higher transport of sediments into downstream channels, suspended in the water column as suspended solids^20^ or settled onto the stream bed, resulting in smothering and homogenization of instream habitats^73,74^. Our data showed that TSS substantially increased after ditch cleaning at TEA, but due to the high variability in the data, it was only statistically significant during high flow periods. Long-term monitoring will be needed to confirm that TSS indeed stabilizes over time in this site where ditch cleaning dug into the fine-textured, mineral soil layer. Background levels of TSS concentrations in forested systems of this region are generally low^75^, but virtually no previous studies exist on sediment transport in waterways that drain catchments affected by forest management operations in Sweden^76^.

### Terrestrial carbon fluxes

The trade-off between reduced CO_2_ but enhanced CH_4_ emissions is a crucial factor determining the carbon sequestration and GHG emission mitigation potential of peatland restoration measures. However, there is currently only limited information existing based on the few published short-term studies in the temperate regions which observed reduced CO_2_ emissions but greatly increased (by up to 20 times) CH_4_ emissions within the first years following restoration in comparison to drained reference sites^8,9,11,77^. Furthermore, the existing studies are all based on upscaling plot-scale flux measurements with a low temporal resolution. To our knowledge this is the first study within the boreal landscape to assess the GHG balance of a rewetted forested peatland based on ecosystem-scale and continuous EC measurements. In contrast to the earlier work, our results suggest that both CO_2_ and CH_4_ emissions increased in the first growing season following restoration. The enhanced CO_2_ emission was likely due to a combination of the eliminated tree photosynthetic CO_2_ uptake and increased decomposition of stumps and roots following tree removal during the restoration work. However, we need to caution on the response magnitude since the Hälsingfors drained forest is more productive (basal area = 16.5 m^2^ ha^−1^) and hence possibly a stronger CO_2_ sink compared to the TEA peatland prior to rewetting. Furthermore, this initial response might be transient and the re-establishment of the CO_2_ sink could be expected within the near future^78,79^. Yet, large uncertainty exists in the trajectories of the CO_2_ and CH_4_ sink-source strength following restoration. The continuation of these measurements will thus be critical to determine the timeframe within which a net climate benefit could be achieved through rewetting of boreal peatlands.

The close-to-zero difference in both NEE and CH_4_ fluxes at the two forest clear-cut sites prior to ditch cleaning suggests that the TEA paired experimental setup will facilitate the evaluation of the treatment effect on GHG fluxes without large confounding effects from pre-treatment differences. The lack of ditch cleaning effects on the difference between NEE and CH_4_ fluxes between the treatment and control sites in the first growing season indicates a relatively slow response of the ecosystem biogeochemistry to this forest management practice. Similarly, Tong et al.^25^ found that ditch cleaning had a limited effect on the NEE in a nearby (~ 30 km) boreal forest clear-cut site during the first 2 years after treatment. However, they found that CH_4_ emissions increased in the control relative to the ditch cleaning area. An initial increase in the CH_4_ uptake following ditch cleaning was also observed in a drained peatland forest clear-cut in hemiboreal Sweden^25^. While initial ditch cleaning effects appear relatively small in both our and these previous studies, gradual changes in soil biogeochemical processes and tree growth in response to ditch cleaning effects on soil water dynamics^3,42,43,80^ are likely to affect carbon fluxes in the long-term. At present, however, our knowledge of ditch cleaning effects on the forest carbon balance is limited to data from a few studies assessing the initial effects based on bi-weekly manual chamber measurements^24,25^. The TEA paired-site experimental setup combining year-round ecosystem-scale EC and spatially distributed plot-level chamber measurements thus offers a unique platform for in-depth ecosystem-scale studies of the ditch cleaning effects on the forest carbon balance in boreal Sweden.

### Integrated findings and ways forward

The question of what to do with the ~ 1 million km of ditches in Sweden requires an integrated perspective that includes the key variables potentially affected by the management alternatives in question. This is important because a specific ditch management action could have positive effects on some key variables, but detrimental effects on others. By including a broad perspective, including the effects on hydrology, a number of water quality variables, and the carbon balance at TEA, we have been able to evaluate several of the essential aspects of three of the most discussed management alternatives of the large ditch network covering much of the boreal landscape.

Any new solution will have to consider how the hydrological conditions will be affected by the new management approach, particularly with regards to the practical issue of why these ditches were dug in the first place—to remove excess water and stimulate tree growth. As shown in our work, any change in the hydrological conditions will result in either saturation of soils or increased drainage. Increasing groundwater levels will activate shallower hydrological pathways, thus, increasing concentrations of DOC, Hg and nutrients, but also increasing the emission of CH_4_, and CO_2_, although for some key elements this effect might be relatively transient. In contrast, decreasing the groundwater levels by ditch cleaning will have the opposite effects, especially for the water quality variables, albeit most changes remained insignificant in the short-term and were on top of a background of increased nutrient exports due to clear-cut. While ditch cleaning in general seems to have had a mitigating influence on the negative effects of the clear-cut for most variables, the 700% increase in sediment load is potentially detrimental to downstream fish habitats and spawning grounds.

One important consideration of this work is the relatively short time-frame over which this study has been conducted. The data from the one to two post-treatment years provide, however, important findings of the initial ecosystem responses to the two management approaches tested but also offer a warning signal for the high uncertainty around the outcomes of these management approaches. Continued monitoring will be necessary to provide a more solid base for future management decisions, and in the meantime, these management decisions should be made more cautiously and carefully given the little published information we have on their outcomes in a Swedish context. While the response time of some of the hydrological effects from both the restoration and ditch cleaning may be within a few weeks–months, other response patterns (i.e. those of the terrestrial C fluxes) can likely take years to several decades before stabilizing. In other cases, the long-term drainage of the catchments could have resulted in alternative environmental states developing, thus, preventing the ecosystems to ever returning to what they once were. Furthermore, crucial aspects to follow up are how long the negative effects from increased greenhouse gas production and mercury export will continue from the restored sites, how long the large sediment load will continue after ditch cleaning, as well as how ditch cleaning could exacerbate drought conditions in a future drier climate. Without lasting commitment to monitoring, it will not be possible to answer questions related to the long-term consequences of these different management actions, and what role they could have to mitigate (or exacerbate) environmental change.

### Supplementary Information

Supplementary Table S1.

## Data Availability

Data on hydrology, water quality and GHG fluxes can be available upon request to the authors.
