# Supplementary material for: Consequences of rewetting and ditch cleaning on hydrology, water quality and greenhouse gas balance in a drained northern landscape
Source: Sci Rep. 2023 Nov 18;13:20218. doi: 10.1038/s41598-023-47528-4 (PMC10657473; doi:10.1038/s41598-023-47528-4)
Supplement: Supplementary file 1 — Supplementary Table S1. [file 41598_2023_47528_MOESM1_ESM.pdf]

7 Supplementary

Table S 1. Catchment characteristics of the Trollberget Experimental Area (TEA). DC1-DC4 are the ditch cleaned experimental sites, where DC1 and DC3 were ditch cleaned and DC2 and DC4 were left alone. R1 and R2 are the hydrological restoration experimental sites.

Catchment	Area (ha)	Peat (%)	Till (%)	Rock outcrops (%)	Type of management	Ditch length (m)	Ditch density (m ha^{-1})	Length of ditches managed (m)	% of ditches managed (%)
DC1	6.7	0	100	0	Ditch cleaning	849	126	658	78
DC2	4.4	0	100	0	Left alone	1117	252	0	0
DC3	8.4	0	100	0	Ditch cleaning	1780	212	1077	60
DC4	10.7	34	63	2	Left alone	795	72	0	0
R1	47	30	43	27	Restoration	1986	42	677	34
R2	60	23	56	22	Restoration	5189	86	824	16
